# Effect of Dioptric Blur on Pattern-Reversal and Motion-Onset VEPs as Used in Clinical Research

**DOI:** 10.1167/tvst.11.12.7

**Published:** 2022-12-06

**Authors:** David Kordek, Petr Voda, Laura K. Young, Jan Kremlacek

**Affiliations:** 1 Department of Biophysics, Faculty of Medicine, Charles University, Hradec Kralove, Czech Republic; 2 Biosciences Institute, Newcastle University, Newcastle, UK

**Keywords:** motion-onset, pattern-reversal, dioptric blur, visual evoked potentials

## Abstract

**Purpose:**

To describe the effect of dioptric blur on visual evoked potentials (VEPs) induced by motion onset (MO-VEPs).

**Methods:**

The effect of dioptric blur up to 4 D on MO-VEPs was tested on 12 subjects using central, peripheral, and full-field stimulation with a low-contrast structure of concentric circles with spatial frequency <1 c/°. The results were compared to VEPs evoked by 15ʹ and 60ʹ checkerboard pattern-reversal (PR-VEPs). The relationship between peak time and interpeak amplitude of the dominant components was related to the level of dioptric blur using linear regression.

**Results:**

The MO-VEPs did not show a significant peak prolongation (*P* > 0.28) or amplitude attenuation (*P* > 0.14) with the blur, whereas for the PR-VEPs we observed a significant decrease in amplitude (*P* < 0.001) and increase in peak time (*P* < 0.001) for both checkerboard sizes.

**Conclusions:**

For MO-VEPs induced by radial motion of low contrast and low spatial frequency pattern, the change in retinal blur does not affect the peak time or the interpeak amplitude of the dominant N2 component.

**Translational Relevance:**

The resistance to retinal blur that we demonstrated for MO-VEP provides a diagnostic opportunity to test the integrity of the visual system and reveal a retrobulbar impairment even in uncorrected refractive errors.

## Introduction

Visual evoked potentials (VEPs) allow noninvasive and inexpensive testing of the integrity of the visual system with high sensitivity and contribute to the diagnosis of retrobulbar pathological processes. The diagnostic parameters of VEPs are influenced by the stimulus's luminance, spatial, and temporal properties. For good inter-laboratory interpretability and high diagnostic yield, stimulus properties are defined by the International Society for the Clinical Electrophysiology of Vision standard.^1^ Because the stimulus is projected onto the retina through the optical environment of the eye, it may be unexpectedly modified at this level. Such modification can cause erroneous inferences about retrobulbar pathology that relate to refractive errors rather than neural effects. For this reason, the VEP examination standard prescribes using at last two stimuli sizes, optimally correcting the eye's refractive error for the stimulus distance, and recording the refractive error in the examination report. The conclusions about the retrobulbar pathology must be supported by an ophthalmological examination of the retina/macula to rule out macular disease.

In some cases, the refractive error cannot be easily corrected (e.g., uncooperative, nonverbal, or malingering subject). It is therefore advantageous to know how the VEP parameters change with artificially blurred vision in individuals whose retrobulbar visual system is intact.^2^^–^^4^

A high degree of dependence of PR-VEP components on the spatial frequency of the stimulus has been demonstrated. Image blurring due to refractive errors leads to a significant increase in the peak time of the P100 component and a decrease in its amplitude^5^^,^^6^ (for review, see^7^).

Thus such high sensitivity of PR-VEP measurements to blurring limits the ability to separate the decrease in visual acuity caused by a refractive error from other impairment. It would be advantageous to use VEP protocols with low sensitivity to refractive errors in this situation. The dominant refractive error, defocus, tends to low-pass filter images formed on the retina, and thus low spatial frequency stimuli are likely to be more robust to refractive errors. Therefore a good candidate for such a task may be motion-onset VEPs (MO-VEPs), which can be evoked by an abrupt motion-onset of low spatial frequency stimuli and even in the periphery and low in contrast.^8^^–^^11^ The dependence of MO-VEPs on artificially induced refractive error has not yet been investigated.

Our study aimed to describe the dependence of MO-VEPs on dioptric blur in the emmetropic eye and compare it to the behavior of PR-VEPs in the same condition. The MO-VEPs showed additional information to pattern-reversal VEPs in neuroborreliosis,^12^^,^^13^ amblyopia,^14^ dyslexia,^15^^,^^16^ glaucoma,^17^ chiasma compression,^18^ and others.^8^^,^^19^ The dominant component of MO-VEP changes significantly with age^20^ and can be elicited by peripheral stimulation^21^^–^^23^ even under scotopic conditions.^24^

The essence of the added diagnostic information is primarily determined by choice of stimulation parameters so that anatomically different parts of the visual system are activated by each type of VEP. The dominant component of the PR-VEP (the P100 wave) is mostly generated in the primary visual cortex^25^^,^^26^ by activation of the central retina^27^ using high contrast and high spatial frequency of reversing structure. The dominant MO-VEP response (wave N2, sometimes N160) is generated in extrastriate areas^28^ associated with spatial perception and attention, i.e., the visual stream “where.”^29^ Stimulus parameters for MO-VEP (low contrast and spatial frequencies) tend to be chosen to activate an input of the magnocellular system predominantly.

## Methods

### Subjects

Twelve men with an age range of 23 to 52 years were examined. The subjects were free of diagnosed neurological and ophthalmological problems at the time of examination. They signed informed consent and data protection consent before the examination. All procedures performed in our study were in accordance with the ethical standards of the institutional committee and with the 1964 Declaration of Helsinki and its later amendments or comparable ethical standards. The ethics committee of the University Hospital in Hradec Kralove approved the study (no. 201411S19P).

### Examination

Examinations were performed in the laboratory of the Department of Pathological Physiology, Faculty of Medicine, Hradec Kralove. Before VEP examination, we measured visual acuity with Landolt optotypes^30^ (at a viewing distance of 4 m), contrast sensitivity with CSV-1000 E (at a viewing distance of 2.5 m, and average luminance 85 cd/m^2^; VectorVision; Guardion Health Sciences Inc., Houston, TX, USA). The refractive state of the eye was measured using an Ark1a refractometer (Nidek, Japan).

During VEP registration, subjects sit comfortably in a darkened electromagnetically shielded room with background ambient light below 1 lux. VEP recording was performed monocularly, and we chose the eye closer to emmetropic vision. If the refractive states of the subject's eyes were identical, we selected the dominant eye, which was identified using the Hole-in-Card Test (Dolman method).

We performed the five VEP examinations, one for each type of stimulation (two pattern reversal and three motion stimuli; see below), and tested each of the five blur conditions during each examination. We pseudo-randomly alternated their order and the blur conditions in each person to control the effect of adaptation and fatigue.^31^^,^^32^ During examination the subject was visually monitored via near infrared camera for correct visual fixation on the stimulus.

### Dioptric Blur

Simulation of refractive blur was performed using external lenses with optical power of 1, 2, 4 D. Because a small dioptric blur can be eliminated by accommodating, we used additional dioptric correction 1.67 D to move the distant point of acuity to the level of the monitor (60 cm).^30^^,^^33^ We also added the individual refractive correction measured with an autorefractometer (RCor). The resulting dioptric power presented in front of each subject's eye and the names of each condition were as follows: RCor (“Corrected”), RCor 1.67D (“Blur 0”), RCor 1.67 + 1D (“Blur 1”), RCor +1.67 + 2D (“Blur 2”), RCor +1.67 + 4D (“Blur 4”).

Because the dioptric test set (Art. 51-BL, M.S.D., Italy) contains lenses of optical power 0.12 D, 0.25, 0.5, 0.75, 1.0, 1.25, 1.5, and more, it was not possible to place a combination of lenses in front of the subject's eye that corresponded to the theoretical dioptric value, except for the condition marked “Corrected.” For the remaining four conditions, the value placed in front of the eye for all subjects was 0.05 D lower than theoretical dioptric value. We used a similar approach in a previous study.^34^

### VEP Stimuli

**Figure 1.**
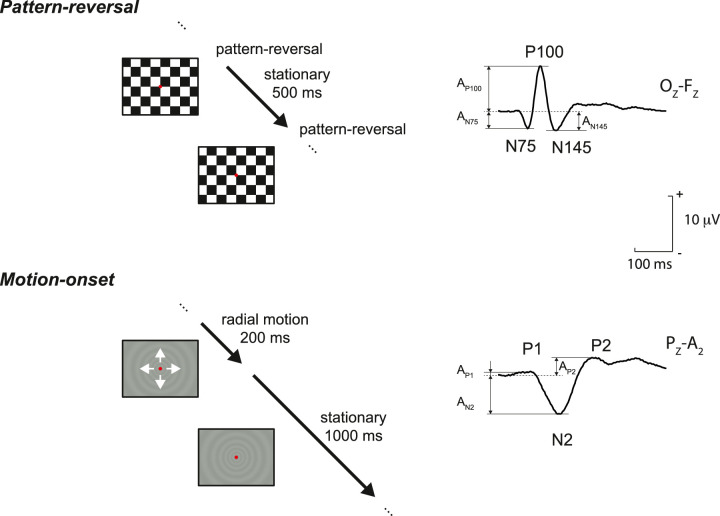
Schematic of the changes for the reversal (top) and motion stimuli (bottom), including a typical VEP waveform in the right part of the figure, where the evaluated parameters of the dominant peaks are marked.

The VEP stimuli were presented on a monitor (Vision Master Pro 510, Iiyama, Japan) with a resolution of 1024 × 768 pixels and an angular size of 37° × 28°, from a viewing distance of 60 cm. Visual stimuli were presented using a Visual Stimulus Generator 2/5 (CRS Ltd., Cheadle, UK) with a vertical refresh rate of 105 Hz. A schematic representation of the PR-VEP and MO-VEP stimulations, the timeline, and the corresponding VEPs, including a typical recording, are shown in Figure 1.

**Figure 2.**
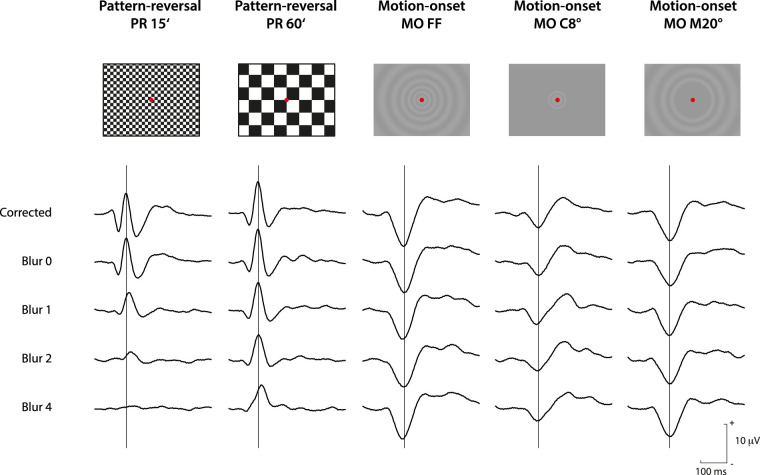
Average VEP recordings for the five stimulations used and five blur conditions. In the upper part of the figure, there is a symbolic depiction of the particular stimulus pattern. From the left, the stimulations are PR 15′, PR 60′, MO FF, MO C8°, MO M20°. Below, the grand average VEPs are created from all subjects for a specific stimulation and blur condition. The effect of blur can be seen in the rows. The changes in the first two columns correspond to the sensitivity of PR-VEPs to dioptric blur. In contrast, the stability of the responses in the right three columns illustrates the robustness of MO-VEPs.

The examination of PR-VEP was based on the International Society for the Clinical Electrophysiology of Vision standard^1^ except for the mean luminance. We used checkerboard stimuli of two elementary square sizes—15 arcmin (Fig. 2, PR 15ʹ) and 60 arcmin (Fig. 2, PR 60ʹ). The Michelson contrast between white and black squares was 96%. The mean luminance was 17 cd/m^2^, less than the standard for PR-VEPs, and was constant for both PR and motion VEPs. The checkerboard pattern spanned the entire area of the monitor and was reversed at a rate of 1 Hz (500 ms per reversal). A red fixation cross was displayed in the center of the monitor during the examination. The single PR-VEP examination lasted 20 seconds and consisted of 40 stimuli.

The MO-VEP reflects the brain's response to the sudden onset of motion in the visual scene. This examination is not standardized, so we used parameters and recommendations of previous studies.^8^^,^^35^^,^^36^ We chose a radial circular pattern with spatial frequency scaled for better visibility in the periphery using a cortical magnification factor (CMF)^36^ defined by the equation:

$$\begin{array}{c} CMF=\frac{1}{0.1E+1}, \end{array}$$

where *E* is the eccentricity in degrees. The spatial frequency was highest in the center of the field of view (1 cycles/°) and decreased toward the periphery (0.2 cycles/°). In motion, the structure reached a velocity of range 5° to 25°/s so that the temporal frequency was 5 Hz. The stationary (1000 ms) and motion periods (200 ms) alternated during the examination. The motion direction was randomly selected as outward (expanding) or inward (imploding) to reduce the motion aftereffect. This pattern was presented: (i) over the entire monitor area (37° × 28° field of view - see Figure 2, MO FF), (ii) in the central 8° (Fig. 2, MO C8°) with the periphery masked, (iii) in the periphery of the monitor with the central 20° masked (Fig. 2, MO M20°). The mean luminance of 17 cd/m^2^ was constant throughout the examination, and maximal Michelson contrast was 10%. The red fixation cross was displayed in the center of the monitor during the examination. A single MO-VEP examination lasted 60 seconds and consisted of 40 stimuli.

### VEP Recording

For the examination of PR-VEP, electroencephalographic activity in the frequency band 0.1 to 100 Hz was sampled at 500 Hz. Post-stimulus epochs of 440 ms were recorded. Epochs with absolute amplitudes greater than 100 mV were excluded from processing. The remaining responses were averaged and smoothed with a second-order Savitzky-Golay filter over 47 samples. The individual discrete values given by the sampling were interpolated for plotting, and any point on the curve could be marked regardless of the sampling frequency when we determined the peaks.

VEPs were recorded using electrodes (OZ, PZ, CZ, FZ, and OL, OR - 5 cm to the left and right of OZ). This laboratory setup included the electrodes standardly used for the PR-VEP (OZ) and MO-VEP (PZ) evaluation. All six channels were recorded and averaged. We did not evaluate electrodes FZ, CZ, OL, and OR. Electrode A2 was used as the ground and reference electrode. The examination was repeated once for all conditions and each type of stimulation. Thus 50 VEPs were obtained for each person.

We read peak time and absolute amplitudes from the PR-VEP for N75, N145, and P100 peaks in Oz-A2 derivation. From the MO-VEP, we read peak time and amplitude values for the P1, N2, and P2 positivity in Pz-A2 derivation (see Fig. 1). Authors D.K. and J.K. performed the evaluation. Peak time values of P100 positivity for PR-VEP and the N2 negativity and their interpeak amplitudes were used for subsequent regression analysis. The interpeak amplitude was the average of the amplitudes measured from the preceding and following peaks for N2 (MO-VEP) and from the preceding and following troughs for P100 (PR-VEP).

### Statistical Analysis

Because two VEPs were registered for each type of stimulation and each level of defocus, the corresponding values were averaged. Using linear regression, we estimated the relationship between averaged parameters and the blur level (Blur 0, Blur 1, Blur 2, and Blur 4) for each person and type of stimulation. In such a way, we got slopes for five types of stimulation and two parameters (peak time and amplitude) in 10 sets. The normality of each set was assessed with the Shapiro-Wilk test. To evaluate the presence of a relationship, we tested whether the set of slopes differed from zero by Student's *t*-test.

## Results

The uncorrected visual acuity of the experimental subjects was in the 4/5 to 4/4 range. The dioptric correction determined by the refractometer ranged from −0.75 to 0.75 D (mean 0.042 ± 0.463 D). All subjects achieved normal visual acuity for a viewing distance of 60 cm with the correction.

**Figure 3.**
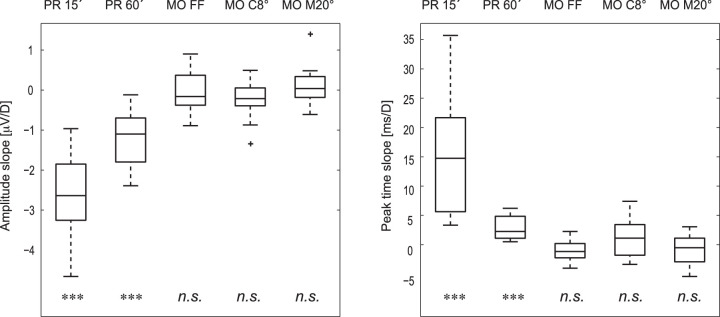
Graphical representation of the distribution of the slopes for PR-VEP and MO-VEP parameters. The slope describes an estimate of how the corresponding parameter changes when the blur increases by 1 D. The left plot shows the slopes for interpeak amplitudes, and the right plot shows those for the peak times. For individual VEPs, the box plots indicate the median and upper and lower quartiles, the dashed lines indicate the 25th and 75th percentiles, and the + sign indicates outliers. The bottom row lists if the slope was statistically different from zero (***P < 0.001, n.s. means nonsignificant). The first two box plots for the PR-VEPs show that the amplitude (left panel) decreases significantly with increasing blur and the peak time (right panel) is prolonged significantly. For MO-VEPs, the slope is insignificant.

Basic descriptive statistics of the peak time and inter-peak amplitude parameters for individual VEP examinations and blur conditions are listed in Table 1. Values for all subjects are presented in the supplementary material (Supplementary Tables S1 and S2). Descriptive statistics of the regression line slopes and the probability of error of rejecting a true null hypothesis for the peak time and interpeak amplitude for PR-VEPs and MO-VEPs are summarized in Table 2 and Figure 3.

**Table 1.** Means and Standard Deviations of the VEP Peak Times

Blur Condition	P100 PR 15ʹ	P100 PR 60ʹ	N2 MO FF	N2 MO C8°	N2 MO M20°
Peak time [ms]					
Corrected	120 ± 5.7	115 ± 4.6	158 ± 11.4	161 ± 11.9	163 ± 11.2
Blur 0	122 ± 5.5	115 ± 6.0	158 ± 8.4	165 ± 13.5	165 ± 15.1
Blur 1	135 ± 8.7	115 ± 6.1	154 ± 9.6	161 ± 12.3	165 ± 12.3
Blur 2	158 ± 26.3	118 ± 6.3	157 ± 12.1	162 ± 18.8	163 ± 10.5
Blur 4	175 ± 35.5	126 ± 8.5	153 ± 9.6	168 ± 10.5	162 ± 8.16
Interpeak amplitude [µV]					
Corrected	11.1 ± 3.6	11.5 ± 3.5	11.4 ± 2.9	6.6 ± 2.6	9.3 ± 2.6
Blur 0	11.0 ± 4.0	11.9 ± 4.0	10.4 ± 2.7	6.4 ± 2.3	8.5 ± 2.4
Blur 1	6.5 ± 2.1	9.7 ± 3.0	10.3 ± 2.7	6.6 ± 2.8	8.6 ± 2.7
Blur 2	2.8 ± 2.0	8.2 ± 2.6	9.6 ± 2.4	5.9 ± 2.2	8.2 ± 3.1
Blur 4	1.5 ± 1.2	6.9 ± 2.0	10.3 ± 3.3	5.6 ± 2.4	9.0 ± 2.4

Means and standard deviations of the peak times (upper part of the table) and interpeak amplitudes (lower part). Values were calculated from the average of two repeated examinations across all subjects for each type of stimulation and level of blur.

**Table 2.** Descriptive Statistic of Regression Line Slopes

	PR 15ʹ	PR 60ʹ	MO FF	MO C8°	MO M20°
Peak time, mean ± SD [ms/D] *P* value	14.83 ± 9.94, 1.6 × 10^−4^	2.92 ± 2.10, 2.8 × 10^−4^	−1.00 ± 1.75, 0.072	1.07 ± 3.51, 0.34	−0.85 ± 2.57, 0.28
Interpeak amplitude, mean ± SD [µV/D] p-value	−2.60 ± 1.01, 1.2 × 10^−6^	−1.20 ± 0.70, 4.8 × 10^−5^	−0.050 ± 0.58, 0.77	−0.24 ± 0.51, 0.14	0.12 ± 0.50, 0.42

The regression line slopes expressing the relationship between the VEP parameters and the level of dioptric blur. The upper part of the table lists the mean and standard deviation of the slopes for the P100 peak time (PR 15’, PR 60’) and for the N2 peak time (MO FF, MO C8°, MO M20°). There are analogous parameters for the slopes of relationship between the interpeak amplitudes and the level of dioptric blur in the lower part. The *P* value represents the *P* value of the one-sample *t*-test of the arithmetic mean of the regression line slope of each parameter compared to zero.

PR-VEP amplitudes reduced sharply with dioptric blur for small checks and more gradually for the large checks (*P* =1.2 × 10^−^^6^ and 4.8 × 10^−^^5^, respectively) with slopes of −2.6 and −1.2 µV per diopter of blur, respectively. P100 peak times increased by 15 ms per diopter of blur for the smaller checks but only by 3 ms per diopter for the larger checks (*P* = 1.6 × 10^−^^4^ and 2.8 × 10^−^^4^, respectively). In the case of MO-VEP, there is neither a significant (*P* ≥ 0.14) change in amplitude nor in N2 peak time (*P* ≥ 0.07) with increasing blur. The regression analysis results for all stimuli, subjects, and parameters are presented in the supplementary material (Supplementary Tables S3 and S4).

## Discussion

Since the 1960s, image blur has been identified as an important factor influencing VEPs. Responses to changes in acuity have been investigated using flashes illuminating a cardboard structure,^37^ by a slide presentation,^38^ or by viewing on a television or computer screen. These experiments were subsequently used for objective selection of refractive correction^39^^–^^41^ and are related to an objective assessment of visual acuity today.^42^

Image quality manipulations can be performed either on the observer side “observer method” or on the image side “source method.”^43^ The “source method” typically uses optical filters near the stimulus (e.g., diffusion foils in front of the screen) or a mathematical function (e.g., convolution of the point spread function with the source pattern^44^) to modify the image as it is displayed. The “observer method” uses an optical device near the observer's eye. We used the latter method and tested how selected parameters of PR-VEPs and MO-VEPs change under dioptric blur.

In our study, as the dioptric blur increased in the PR-VEP experiments, the interpeak amplitude of PR-VEP Amplitude decreased, and the peak time of the dominant P100 wave was prolonged. Similar observations for the PR-VEP have already been published. Individual studies have reported significant results for small structure sizes and higher blur levels, but they vary in the degree of amplitude decrease and peak time prolongation.^45^^–^^48^ The variability in effect size can be attributed to inter-study uncontrolled factors such as the method of image defocus, the magnitude of brightness and contrast of the stimulus used, and the individual subject factors such as age, pupil size, cortical shape, and more. A visual enhancement using adaptive optics has also produced similar outcomes. After correcting for higher-order aberrations, Yang et al.^49^ reported a statistically significant increase in the amplitude of the positive P100 peak for frequencies 1- 16 cycles/°.

The interpeak amplitude and peak time of the dominant peak N2 of VEPs tied to the onset of visual motion remained without statistically significant changes in our study. Although the response stability to retinal blur is a novel finding, it was described that MO-VEPs were independent of the spectral content of the stimulus pattern.^50^

The robustness of the MO-VEP to blur in our study could arise from various factors. One is the stimulus pattern resistance to the dioptric blur. The MO-VEP stimulus pattern is essentially a set of low-frequency sinusoidal gratings presented as concentric rings. It was designed to activate the magnocellular input of the visual analyzer, which is effective even at low contrasts and detects low spatial frequencies because of large receptive fields.^51^ Dioptric blur acts like a low pass filter and attenuates contrast of higher components of the stimulus frequency spectrum.^52^ For our setup, the dioptric blur had a negligible impact on the MO-VEP stimulus spectrum.

**Figure 4.**
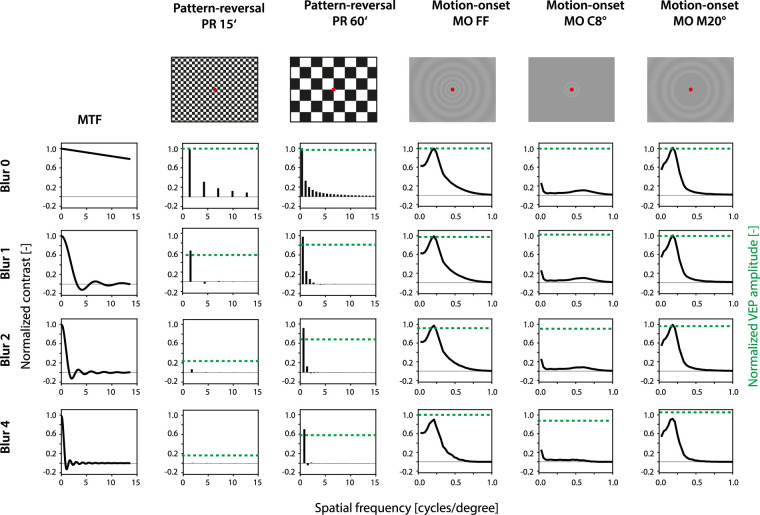
The modulation transfer functions (MTFs) for a diffraction-limited eye (MTF for Blur 0) and the refractive errors introduced (Blur 1–4) are displayed in the first column. The rest of the image illustrates stimulus appearances (the first row) and their Fourier power spectra multiplied with the appropriate MTFs (rows 2–5) as they are formed on the retina for a given refractive error. The second and the third columns show the PR-VEP stimuli, essentially square waves containing a fundamental frequency and odd harmonics. The blur strongly modified the power spectrum of the checkerboard. The fourth to sixth columns show the power spectra of the MO-VEP stimuli, demonstrating minimal modification by the refractive errors introduced. Note the different horizontal scale for MO-VEPs versus PR-VEP stimuli. The green dashed lines show the normalized VEP amplitude reduction for the given dioptric blur. Refractive errors were modeled for a pupil diameter of 5 mm and a wavelength of 550 nm. It should be noted that contrast reversals occur in particular spatial frequency bands, which is equivalent to a 180° phase shift. The two-dimensional spectrum was derived analytically for the PR 16′, PR 60′ based on a slice in the diagonal direction. The motion-onset spectra show the result of a numerical transformation of the two-dimensional stimulus image, collapsed into a radial profile. The MTF is also computed numerically, using the specified point spread function, and collapsed to a radial profile.

On the other hand, the spatial frequency spectrum of the PR-VEP stimulus contains high frequencies because of the sharp edges between the dark and light fields, constituting the checkerboard. The power spectrum of the PR-VEP stimulus is much more affected by the introduction of a refractive error than the MO-VEP stimulus, especially for the small checks. Figure 4 visualizes the effect. The modulation transfer function of an optical system that is either diffraction-limited or has 1, 2, or 4 D of defocus we used to modify the spatial frequency content of stimuli.

The attenuation of high spatial frequencies alone does not fully explain the robustness of MO-VEPs to the blur. Dioptric blur brought the PR-VEP stimulus frequency content closer to the MO-VEP pattern (especially for PR 60ʹ) by reducing the contrast of the harmonics, as demonstrated in Figure 4 (Note that it is impossible to compare the relative contrast between PR-VEP and MO-VEP stimuli in Figure 4, thanks to the relative scales and different entry contrasts.)

Still, the PR-VEP deteriorated contrary to the MO-VEP response. The most probable reason for M-VEPs resistance to the dioptric blur is their insensitivity to contrast changes contrary to the PR-VEP. Dioptric blur can be characterized as a drop in contrast of high spatial frequencies in the retinal image.^52^ The two types of VEPs we examined respond differently to contrast changes. The amplitude of the dominant component of the MO-VEP does not change with stimulus contrast until it drops to 2.3%. However, the amplitude of the dominant component of the PR-VEP decreases from a contrast as high as 64%.^11^^,^^53^

In our experiment, we evaluated the effect of dioptric blur on low luminance PR-VEPs similar to those used in clinical testing. A next step to get more-specific responses about the relationship of MO-VEP and PR-VEP with respect to the retinal blur one could use the same stimulation structure for both VEP types, use the source-side blur method to suppress variability in the blur level, and vary the temporal frequency systematically. The exact comparison of both VEP types will not be as easy, because both VEPs have different cortical origins, contrast, and spatial frequency sensitivity.^11^^,^^53^

### Limitations

Use of dioptrical blurring on the observer's side is burdened by variability in vertex distance or looking off the lens's optical axis. We kept the vertex distance constant and checked the head and the eye position during the VEP examination via a NIR video camera to eliminate these effects. Furthermore, because the intraindividual variability is much lower than the dioptric blur changes, the conclusions about the different behavior of PR-VEP and MO-VEP in the high blur values are reliable.

Our study does not provide information on how MO-VEPs will be affected by higher-order refractive errors. To test the effect of these deformations, using rendered aberrations on the source side would be practical. However, similar to the spherical error, we expect that the influence of higher-order aberrations will have a minimal effect on the MO-VEPs because the higher-order aberrations mainly affect higher spatial frequencies.^54^

Pupil size negatively correlates with PR-VEP peak time and amplitude.^55^ In our experiment, the most significant factors, which may modulate pupil size (luminance and viewing distance), were the same across all conditions. Moreover, PR-VEP peak time fluctuations caused by the different pupil size could be in a range of single milliseconds.^56^ We measured P100 peak time changes of 55 ms for R15 and 11 ms for R60 due to dioptric blur (see Table 1). Thus it is unlikely that different pupil sizes caused the changes in VEPs we observed.

## Conclusion

In this experiment, we have shown that optical defocusing with a lens of up to 4 D does not induce elongation or reduction of the dominant component of the VEP induced by motion-onset of low contrast, low temporal frequency stimuli in the visual field. In contrast, VEPs elicited by pattern-reversal stimulation are significantly altered by this defocus.

## Supplementary Material

Supplement 1
