# Supplementary material for: Effect of Dioptric Blur on Pattern-Reversal and Motion-Onset VEPs as Used in Clinical Research
Source: Transl Vis Sci Technol. 2022 Dec 6;11(12):7. doi: 10.1167/tvst.11.12.7 (PMC9733653; doi:10.1167/tvst.11.12.7)
Supplement: Supplement 1 [file tvst-11-12-7_s001.docx]

**Effect of dioptric blur on pattern-reversal and motion onset VEPs**

D. Kordek^1^. P. Voda^1^. L. K. Young^2^. J. Kremláček^1^

^1^Department of Biophysics. Faculty of Medicine. Charles University. Hradec Kralove. Czech Republic

^2^Biosciences Institute. Newcastle University. Newcastle. UK

Corresponding author: Jan Kremlacek. Ph.D. (jan.kremlacek@lfhk.cuni.cz)

Table S1 – Peak time values [ms] for P100 positivity (for PR VEP) and N2 negativity (for MO VEP). Each value in the table is always the arithmetic mean of two measured values for each stimulation and for each dioptric blur value.

| subject | blur | P100 PR 15´ | P100 PR 60´ | N2 MO FF | N2 MO C8° | N2 MO M20° |
| --- | --- | --- | --- | --- | --- | --- |
| S001 | 0 abs | 119 | 121 | 165 | 166 | 168 |
|  | 0 | 119 | 118 | 150 | 170 | 162 |
|  | 1 | 141 | 120 | 149 | 169 | 156 |
|  | 2 | 167 | 125 | 163 | 176 | 158 |
|  | 4 |  | 125 | 157 | 175 | 163 |
| S002 | 0 abs | 109 | 114 | 168 |  | 183 |
|  | 0 | 116 | 117 | 166 |  | 184 |
|  | 1 | 154 | 115 | 167 |  | 180 |
|  | 2 | 162 | 115 | 165 |  |  |
|  | 4 |  | 139 | 171 |  | 180 |
| S003 | 0 abs | 122 | 115 | 166 | 176 | 143 |
|  | 0 | 120 | 114 | 148 | 185.5 | 146 |
|  | 1 | 126 | 108 | 143 | 182 | 176 |
|  | 2 |  | 110 | 138 | 196 | 154 |
|  | 4 | 134 | 116 | 137 | 170 | 156 |
| S004 | 0 abs | 118 | 112 | 171 | 168 | 167 |
|  | 0 | 119 | 112 | 167 | 180 | 181 |
|  | 1 | 128 | 113.5 | 171 | 181 | 172 |
|  | 2 | 226 | 114 | 164.5 | 168 | 178 |
|  | 4 | 249 | 115 | 153 | 177 | 162 |
| S005 | 0 abs | 117 | 109 | 148 | 162 | 163 |
|  | 0 | 116 | 107 | 158 | 162 | 147 |
|  | 1 | 128 | 109 | 152 | 161 | 150 |
|  | 2 | 135 | 111 | 153 | 177 | 157 |
|  | 4 | 183 | 121 | 148 | 182 | 153 |
| S006 | 0 abs | 125 | 113 | 164 | 170 | 170 |
|  | 0 | 127 | 118 | 171 | 181 | 178 |
|  | 1 | 136 | 115 | 170 | 163 | 167 |
|  | 2 | 142 | 119 | 179 | 170 | 170 |
|  | 4 | 158 | 125 | 167 | 166 | 172 |
| S007 | 0 abs | 114 | 112 | 131 | 133 | 146 |
|  | 0 | 117 | 113 | 153 | 167 | 147 |
|  | 1 | 122 | 113 | 147 | 156 | 168 |
|  | 2 | 133 | 120 | 153 | 154 | 170 |
|  | 4 | 196 | 135 | 152 | 157 | 158 |
| S008 | 0 abs | 118 | 113 | 159 | 149 | 153 |
|  | 0 | 121 | 112 | 149 | 148 | 147 |
|  | 1 | 136 | 114 | 151 | 147 | 143 |
|  | 2 | 171 | 119 | 147 | 149 | 143 |
|  | 4 | 170 | 136 | 141 | 162 | 158 |
| S009 | 0 abs | 117 | 114 | 168 | 163 | 171 |
|  | 0 | 123 | 114 | 170 | 158 | 180 |
|  | 1 | 133 | 115 | 152 | 153 | 179 |
|  | 2 | 136.5 | 116 | 168 | 159 | 176 |
|  | 4 |  | 116 | 158 | 185 | 159 |
| S010 | 0 abs | 125 | 111 | 151 | 160.5 | 158 |
|  | 0 | 126 | 109 | 159 | 145.5 | 162 |
|  | 1 | 135 | 113 | 152 | 146 | 154 |
|  | 2 | 153 | 114 | 157 | 127 | 156 |
|  | 4 | 163 | 125 | 152 | 152 | 150 |
| S011 | 0 abs | 126 | 115 | 154 | 152 | 167 |
|  | 0 | 127 | 115 | 154 | 152 | 164 |
|  | 1 | 143 | 117 | 149 | 152 | 160 |
|  | 2 | 162 | 116 | 152 | 143 | 160 |
|  | 4 |  | 125 | 153 | 163 | 162 |
| S012 | 0 abs | 129 | 126 | 153 | 166 | 162 |
|  | 0 | 134 | 131 | 153 | 166.5 | 177 |
|  | 1 | 139 | 132 | 146 | 159 | 176 |
|  | 2 | 147 | 133 | 139 | 157 | 166 |
|  | 4 | 149 | 136 | 148 | 161 | 165 |

Table S2 – Interpeak amplitude values [µV] PR-Am = (P100_amplitude_ - N75 _amplitude_)/2 + (P100 _amplitude_ - N145 _amplitude_)/2 (u PR VEP) a MO-Am = (P1 _amplitude_ - N2 _amplitude_)/2 + (P2 _amplitude_ - N2 _amplitude_)/2. Each value in the table is always the arithmetic mean of two measured values for each stimulation and for each dioptric blur value.

| subject | blur | PR 15´ | PR 60´ | MO FF | MO C8° | MO M20° |
| --- | --- | --- | --- | --- | --- | --- |
| S001 | 0 abs | 10.2825 | 7.6125 | 6.98 | 5.405 | 4.34 |
|  | 0 | 9.6225 | 7.4725 | 8.535 | 6.915 | 7.71 |
|  | 1 | 4.8175 | 6.945 | 9.0925 | 6.2675 | 6.08 |
|  | 2 | 0.305 | 6.74 | 7.4725 | 5.315 | 8.62 |
|  | 4 | 0 | 5.68 | 6.7675 | 3.49 | 6.63 |
| S002 | 0 abs | 2.6575 | 6.8125 | 8.78 | 5.7825 | 7.39 |
|  | 0 | 3.0325 | 7.5075 | 9.7675 | 8.7425 | 7.27 |
|  | 1 | 3.9475 | 5.5425 | 8.0675 | 3.3425 | 5.94 |
|  | 2 | 1.1 | 3.4975 | 5.5175 | 3.3175 | 1.17 |
|  | 4 | 0 | 2.55 | 6.2125 | 2.19 | 7.28 |
| S003 | 0 abs | 12.625 | 10.5325 | 9.845 | 3.365 | 10.83 |
|  | 0 | 15.1125 | 11.545 | 8.395 | 5.785 | 9.81 |
|  | 1 | 9.145 | 8.445 | 9.08 | 4.085 | 9.25 |
|  | 2 | 0 | 9.13 | 9.575 | 4.625 | 8.26 |
|  | 4 | 1.24 | 7.4675 | 11.3775 | 3.3325 | 10.01 |
| S004 | 0 abs | 11.87 | 12.6725 | 11.3425 | 7.85 | 7.78 |
|  | 0 | 13.205 | 13.7225 | 12.5625 | 7.985 | 5.63 |
|  | 1 | 4.785 | 10.165 | 10.4025 | 6.9625 | 6.75 |
|  | 2 | 1.38 | 8.2625 | 10.4 | 8.4425 | 7.12 |
|  | 4 | 3.23 | 7.8325 | 12.085 | 6.72 | 7.33 |
| S005 | 0 abs | 13.93 | 14.905 | 12.8725 | 9.35 | 8.93 |
|  | 0 | 16.455 | 15.6775 | 10.5525 | 6.03 | 11.66 |
|  | 1 | 9.585 | 13.3325 | 12.9225 | 7.97 | 10.94 |
|  | 2 | 6.8075 | 10.3975 | 10.995 | 4.805 | 10.42 |
|  | 4 | 2.275 | 7.63 | 10.6475 | 6.4625 | 9.18 |
| S006 | 0 abs | 9.94 | 10.01 | 16.4 | 10.41 | 12.48 |
|  | 0 | 9.5 | 11.4 | 15.26 | 7.9725 | 9.17 |
|  | 1 | 8.455 | 8.6025 | 14.9675 | 12.2 | 10.35 |
|  | 2 | 4.2425 | 9.145 | 12.7025 | 9.6025 | 12.07 |
|  | 4 | 2.41 | 7.4425 | 14.72 | 9.8 | 10.36 |
| S007 | 0 abs | 11.4275 | 10.7975 | 8.72 | 5.8175 | 7.18 |
|  | 0 | 8.9525 | 10.3075 | 8.59 | 2.53 | 6.94 |
|  | 1 | 5.9 | 11.39 | 7.725 | 5.2075 | 6.11 |
|  | 2 | 4.375 | 6.4675 | 6.0325 | 4.5875 | 4.61 |
|  | 4 | 2.9725 | 8.9375 | 5.535 | 4.55 | 5.92 |
| S008 | 0 abs | 11.7625 | 11.205 | 9.06 | 2.5025 | 8.07 |
|  | 0 | 12.4525 | 11.485 | 5.805 | 3.3025 | 4.94 |
|  | 1 | 6.655 | 9.7 | 6.695 | 4.225 | 6.17 |
|  | 2 | 2.77 | 7.445 | 8.9025 | 2.49 | 6.98 |
|  | 4 | 1.5 | 4.97 | 8.6775 | 2.9075 | 7 |
| S009 | 0 abs | 6.405 | 9.5475 | 11.105 | 6.47 | 8.76 |
|  | 0 | 6.4225 | 9.005 | 9.515 | 5.4725 | 6.37 |
|  | 1 | 5.5775 | 7.76 | 8.285 | 6.405 | 7.47 |
|  | 2 | 2.7825 | 6.4675 | 8.5825 | 6.5125 | 7.08 |
|  | 4 | 0 | 5.0675 | 8.5975 | 5.485 | 7.31 |
| S010 | 0 abs | 12.4625 | 16.08 | 12.11 | 3.9275 | 12.44 |
|  | 0 | 13.7375 | 18.715 | 10.5875 | 4.0725 | 11.87 |
|  | 1 | 8.025 | 13.2725 | 10.7575 | 3.285 | 12.64 |
|  | 2 | 3.56 | 12.6475 | 10.1125 | 4.9025 | 11.03 |
|  | 4 | 2.5425 | 9.9575 | 9.8175 | 5.63 | 11.13 |
| S011 | 0 abs | 13.045 | 18.7075 | 15.11 | 8.5425 | 12.57 |
|  | 0 | 8.3925 | 18.23 | 14.7025 | 9.745 | 11.86 |
|  | 1 | 3.625 | 14.5925 | 14.4775 | 9.665 | 13.41 |
|  | 2 | 1.9325 | 11.825 | 13.315 | 7.8925 | 11.99 |
|  | 4 | 0 | 8.42 | 14.5775 | 8.8775 | 12.44 |
| S012 | 0 abs | 16.2125 | 8.8075 | 14.7025 | 9.38 | 10.82 |
|  | 0 | 14.755 | 7.5325 | 10.9575 | 8.4275 | 8.21 |
|  | 1 | 7.9025 | 6.0125 | 10.85 | 9.2525 | 7.81 |
|  | 2 | 4.8775 | 6.7275 | 11.1475 | 8.2225 | 8.69 |
|  | 4 | 1.415 | 6.65 | 14.405 | 7.815 | 13.33 |

Table S3 – [ms/D] - peak time - the slopes of the regression lines for P100 positivity (PR VEP) and N2 negativity (MO VEP)

|  | subject | MO FF | MO C8° | MO M20° | PR 15´ | PR 60´ |
| --- | --- | --- | --- | --- | --- | --- |
| the slopes of the regression lines | S001 | 0.26 | -1.20 | -3.31 | 35.71 | 6.20 |
|  | S002 | -2.20 | -2.00 | -4.20 | 20.34 | 3.91 |
|  | S003 | -2.29 | -3.39 | -5.43 | 16.74 | 5.77 |
|  | S004 | 2.26 | 1.54 | -0.83 | 12.74 | 2.09 |
|  | S005 | -1.60 | 2.57 | 1.57 | 9.51 | 2.43 |
|  | S006 | -2.69 | -1.04 | -0.29 | 3.80 | 5.86 |
|  | S007 | -0.71 | 7.40 | 3.06 | 4.51 | 0.49 |
|  | S008 | 0.11 | 1.10 | 0.23 | 3.31 | 1.83 |
|  | S009 | -1.26 | 3.71 | 1.69 | 6.75 | 3.54 |
|  | S010 | -1.09 | 5.66 | 0.66 | 24.00 | 1.26 |
|  | S011 | 1.17 | -2.63 | -2.57 | 17.50 | 0.91 |
|  | S012 | -4.01 |  | -0.77 | 23.00 | 0.70 |

Table S4 – [µV/D] - interpeak amplitude - the slopes of the regression lines for **PR-Am** = (P100_amplitude_ - N75 _amplitude_)/2 + (P100 _amplitude_ - N145 _amplitude_)/2 (u PR VEP) and **MO-Am** = (P1 _amplitude_ - N2 _amplitude_)/2 + (P2 _amplitude_ - N2 _amplitude_)/2.

|  | subject | MO FF | MO C8° | MO M20° | PR 15´ | PR 60´ |
| --- | --- | --- | --- | --- | --- | --- |
| the slopes of the regression lines | S001 | -0.5326 | -1.3771 | -0.112 | -3.3331 | -1.9588 |
|  | S002 | 0.0006 | -0.5181 | -0.6114 | -2.6799 | -0.8423 |
|  | S003 | -0.8899 | -0.8709 | 0.2877 | -0.9663 | -1.3656 |
|  | S004 | -0.7845 | -0.2691 | 0.02 | -1.8838 | -2.0192 |
|  | S005 | -0.1869 | -0.2039 | 0.4826 | -3.1251 | -1.6377 |
|  | S006 | 0.7419 | 0.4917 | -0.2803 | -1.4069 | -0.4366 |
|  | S007 | -0.1571 | -0.2341 | 1.3646 | -3.23 | -2.3938 |
|  | S008 | -0.1661 | 0.1541 | -0.2577 | -1.82 | -0.1196 |
|  | S009 | 0.7509 | 0.3487 | 0.3837 | -2.5961 | -0.9783 |
|  | S010 | 0.9011 | -0.047 | 0.1677 | -2.1811 | -0.5548 |
|  | S011 | -0.2261 | -0.0901 | -0.0577 | -4.6588 | -0.8518 |
|  | S012 | -0.0525 | -0.2246 | 0.0551 | -3.2759 | -1.2209 |
